# ACAA2 is a novel molecular indicator for cancers with neuroendocrine phenotype

**DOI:** 10.1038/s41416-023-02448-y

**Published:** 2023-10-05

**Authors:** Michelle Shen, Shiqin Liu, Angus Toland, En-Chi Hsu, Alifiani B. Hartono, Busola R. Alabi, Merve Aslan, Holly M. Nguyen, Conner J. Sessions, Rosalie Nolley, Chanjuan Shi, Jiaoti Huang, James D. Brooks, Eva Corey, Tanya Stoyanova

**Affiliations:** 1 Department of Radiology, Stanford University, Stanford, CA USA; 2 Canary Center at Stanford for Cancer Early Detection, Stanford University, Stanford, CA USA; 3 Department of Molecular and Medical Pharmacology, University of California Los Angeles, Los Angeles, CA USA; 4 Department of Pathology, Stanford University, Stanford, CA USA; 5 Department of Urology, University of Washington, Seattle, WA USA; 6 Department of Urology, Stanford University, Stanford, CA USA; 7 Department of Pathology, Duke University, Durham, NC USA; 8 Department of Urology, University of California Los Angeles, Los Angeles, CA USA

**Keywords:** Tumour biomarkers, Prostate cancer, Small-cell lung cancer

## Abstract

**Background:**

Neuroendocrine phenotype is commonly associated with therapy resistance and poor prognoses in small-cell neuroendocrine cancers (SCNCs), such as neuroendocrine prostate cancer (NEPC) and small-cell lung cancer (SCLC). Expression levels of current neuroendocrine markers exhibit high case-by-case variability, so multiple markers are used in combination to identify SCNCs. Here, we report that ACAA2 is elevated in SCNCs and is a potential molecular indicator for SCNCs.

**Methods:**

ACAA2 expressions in tumour xenografts, tissue microarrays (TMAs), and patient tissues from prostate and lung cancers were analysed via immunohistochemistry. *ACAA*2 mRNA levels in lung and prostate cancer (PC) patients were assessed in published datasets.

**Results:**

ACAA2 protein and mRNA levels were elevated in SCNCs relative to non-SCNCs. Medium/high ACAA2 intensity was observed in 78% of NEPC PDXs samples (*N* = 27) relative to 33% of adeno-CRPC (*N* = 86), 2% of localised PC (*N* = 50), and 0% of benign prostate specimens (*N* = 101). ACAA2 was also elevated in lung cancer patient tissues with neuroendocrine phenotype. 83% of lung carcinoid tissues (*N* = 12) and 90% of SCLC tissues (*N* = 10) exhibited medium/high intensity relative to 40% of lung adenocarcinoma (*N* = 15).

**Conclusion:**

ACAA2 expression is elevated in aggressive SCNCs such as NEPC and SCLC, suggesting it is a potential molecular indicator for SCNCs.

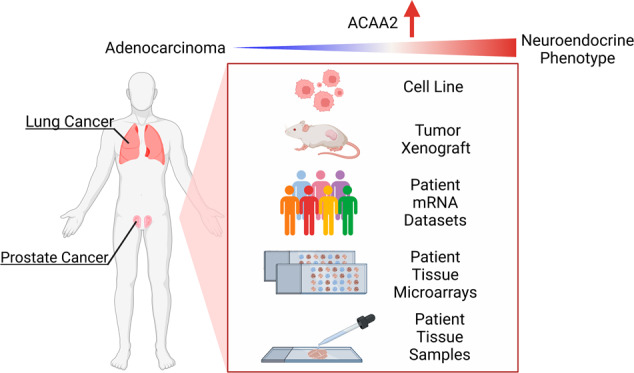

## Introduction

Neuroendocrine (NE) phenotype in lung and prostate cancer frequently correlates with an aggressive clinical course, therapy resistance, and widespread metastasis, which contributes to worse clinical outcomes [1, 2]. For instance, neuroendocrine prostate cancer (NEPC) is the most lethal subtype of prostate cancer, and small-cell lung cancer (SCLC) is also an aggressive, highly lethal subtype of lung cancer [3]. Studies have demonstrated that these small-cell neuroendocrine cancers (SCNCs) from different tissues are more similar to each other than to adenocarcinomas from the same tissue site despite differences in the tissue of origin [4]. They share many histopathological commonalities in morphology, such as high nuclear-to-cytoplasm ratios, poorly defined borders, and granular chromatin [1, 5]. SCNCs also share common gene alterations and an expression of a common set of markers, including synaptophysin (SYP), chromogranin A (CHGA), and CD56, suggesting common drivers and transdifferentiating pathways [1, 5, 6].

The cell of origin of SCNCs remains unclear. Previous studies observed that tumours containing both NE and adenocarcinoma features display an increase of NE phenotype over time during disease progression and the onset of treatment resistance, thereby suggesting that NE transdifferentiation may arise from adenocarcinoma precursors [1, 6, 7]. SCNCs such as NEPC and SCLC can arise from heavily treated adenocarcinoma via cancer’s adaptive response, increased stemness, and lineage plasticity, which enhances therapy resistance [8–10]. For instance, once castration-resistant prostate adenocarcinoma (adeno-CRPC) gains NE phenotype and advances to NEPC during intensive treatment with a new generation of anti-androgen therapies, its median survival decreases to about 7 months, and these diseases are resistant to conventional anti-androgen therapies due to loss of dependence on AR signalling pathways [10–16].

Current clinical identification of SCNCs relies on morphological characteristics and histological markers such as SYP, CHGA, and CD56. However, the expression of these markers varies based on the patient, which limits the reliability of any single histological markers [4, 17]. Thus, multiple markers must be used in combination to effectively assess the presence of NE phenotypes since SCNC tumours can express various profiles of NE markers [4, 17]. In addition, the search for common molecular indicators across SCNCs may potentially lead to the discovery of new NE driver pathways, precision oncology, and new targeted therapies [18].

A common transdifferentiation across SCNCs suggests mutual vulnerabilities and treatment targets, rendering a shared targeted therapy across SCNCs a possibility [6, 19, 20]. We hypothesise that uncovering the common oncogenic pathways in SCNCs fosters the identification of common therapeutic targets across these aggressive tumours [19]. Therapies targeting mutual pathways of SCNCs, such as Myc-targeting Aurora kinase inhibition [21–23] and EZH2 inhibition (NCT03460977; NCT03480646), are currently being explored in NEPC, SCLC, and other NE cancers [1, 24, 25].

To identify new molecular indicators and therapeutic targets for SCNCs, we analysed a previously published proteomic dataset containing SCNC and non-SCNC tumours [26]. We demonstrated that Acetyl-CoA acyltransferase 2 (ACAA2) is highly upregulated in a Trop2-driven NEPC (TD-NEPC) model [26]. ACAA2, also known as 3-ketoacyl-CoA thiolase, is a rate-limiting enzyme in the mitochondria that is responsible for catalysing the last step of the mitochondrial beta-oxidation pathway [27–29]. ACAA2 is associated with cardiovascular risks and lipid metabolism, but its role in cancer has not been fully elucidated [30–33]. A previous study suggests that SCNCs are susceptible to disruptions of genes in the lipid metabolism pathway through genome-wide functional RNA interference screens [4]. Here, we report that ACAA2 expression is increased in SCNCs relative to non-SCNCs in cell lines, tumour xenografts, and patient transcriptomic datasets, suggesting ACAA2 as a potential molecular indicator for these malignancies.

## Materials and methods

### Cell culture

The human prostate cancer cell lines used, including LNCaP, C4-2, 22RV1, DU145, PC3, ARCaP, and NCI-H660 were purchased from the American Type Culture Collection (ATCC). Castration-sensitive prostate cancer (CSPC) cell line, LNCaP, was used to overexpress Trop2 to generate the Trop2-driven NEPC (TD-NEPC) as described in Hsu et al. (2020) [26]. LNCaP, C4-2, 22RV1, DU145, PC3, ARCaP, and TD-NEPC cells were maintained in RPMI 1640 medium (Thermo Fisher Scientific), which was supplemented with 10% FBS, 100 U/ml penicillin, and 100X GlutaMAX. NCI-H660 cells were cultured in RPMI 1640 medium with 5% fetal bovine serum, 0.005 mg/ml insulin, 0.01 mg/ml transferrin, 30 nM sodium selenite, 10 nM hydrocortisone, 10 nM beta-estradiol, 4 mM L-glutamine.

### Western blot (WB)

Cells were collected from culture and lysed using RIPA lysis buffer with protease and phosphatase inhibitors (Thermo Fisher Scientific). BCA assay was performed to measure protein concentration of the lysate, and equal amounts of protein (40 μg/20 μl) were added for each sample. SDS buffer was added to samples, and heat denaturation was performed at 95 °C for 5 min. Samples were loaded and separated by 8–16% SDS-PAGE gel (Invitrogen™ XP08165BOX), transferred onto a 0.22 μm nitrocellulose membrane (GVS Life Sciences, 1212632), and blocked for an hour under room temperature with 5% non-fat milk. The blocked membrane was then incubated with primary antibodies overnight at 4 °C. Anti-ACAA2 was purchased from Abcam (ab128929, 1:1000 dilution), and GAPDH (sc-47724, 1:2000 dilution) was used as an internal control. After overnight incubation, blots were washed with TBST 3 times, 5 min each. Then, blots were incubated for an hour in secondary antibodies, purchased from Fisher Scientific, with HRP conjugation (PI31432 and PI31462, 1:5000 dilution). The signal was detected using PierceTM ECL Western Blotting Substrate (Thermo Fisher Scientific).

### Xenografts

For all the prostate cancer cell lines, 10^6^ cells were mixed with Matrigel (100 μL) and implanted into the flanks of 6-to-8-weeks old male NSG (NOD-SCID-IL2Rγ-null) mice via subcutaneous injection. Xenografts were then harvested from these mice, fixed, and paraffin-embedded for sectioning into 4-microns thick sections with Epredia™ HM 355S Automatic Microtome.

### Animals

All animal work was performed in accordance with protocols approved by the Institutional Animal Care and Use Committee (IACUC) at Stanford University, the USAMRMC Animal Care and Use Review (ACURO), and the laws and regulations of the Department of Agriculture in the United States.

### Immunohistochemistry (IHC)

All tumours used for IHC were sectioned to 4 microns in thickness from formalin-fixed, paraffin-embedded tissues. The antibody used for ACAA2 staining was purchased from Abcam (ab128929, 1:100 dilution). The antibody used for CHGA staining was purchased from Santa Cruz Biotechnology (sc-393941, 1:100 dilution). The sections were deparaffined for an hour in a heated chamber at 65 °C. Then, rehydrated in Clearify for 15 min, 100% ethanol for 10 min, 95% ethanol for 10 min, 70% ethanol for 5 min, and water for 10 minutes. Then, antigen retrieval was performed by immersing the slides in 95 °C, 10 mM citrate buffer (pH = 6.0) for 30 min. After cooling the slides to room temperature, tissues were covered in 3% hydrogen peroxide for 5 min to block endogenous peroxidase activity. Slides were then blocked with 2.5% horse serum diluted in 1xPBS for an hour in a humidified chamber under room temperature. After blocking, sections were incubated overnight with biotin-conjugated primary anti-ACAA2 antibody at 4 °C (1:100 dilution in blocking solution). The next day, slides were washed three times with 1XPBS and incubated with streptavidin-horseradish peroxidase (SA-5004, 1:200, Vector Laboratories) for an hour at room temperature. After washing the slides three times in 1XPBS again, a DAB substrate kit (Dako, as per the manufacturer’s protocol) was used to visualise the staining signal. The sections were then stained with hematoxylin and dehydrated. After mounting and adding coverslips to the slides, all slides were scanned using a NanoZoomer (Hamamatsu).

### Tissue microarrays (TMAs) and patient tissue samples

The patient TMAs with benign and localised prostate cancer cores was constructed at Stanford University, Department of Urology. ACAA2 staining on these TMAs included 35 patients with benign prostate (101 cores) and 18 patients with localised prostate cancer (50 cores). All sample collection was approved by the Institutional Review Board (IRB), and informed consent was provided by all patients under the protocol number IRB: 5628. The LuCaP prostate cancer patient-derived xenograft (PDX) TMA was constructed at the University of Washington. 38 models are present on this TMA, with three cores of three different tumours of each model. ACAA2 staining on this PDX TMAs included 29 adeno-CRPC PDXs with 86 cores and 9 NEPC PDXs with 27 cores. Cores that were damaged or lost during IHC staining were excluded. 10 SCLC, 12 lung carcinoid, and 15 lung adenocarcinoma patient tumour samples were purchased from the Stanford Cancer Institute (SCI) Tissue Bank, and the diagnoses were validated by pathologists. Informed consent was provided by all patients for sample collection under the approved IRB protocol (IRB: 11977). ACAA2 staining in 20 NE neoplasms from 9 organs (mediastinum, cardia, gallbladder, colon, small intestine, pancreas, rectum, stomach, and lung), 16 adenocarcinomas, and 8 normal tissues were performed on an TMA (2 cores per case) purchased from tissuearray.com (#NE921). All staining was scored without awareness of patient clinical information on an intensity-based scale from 0 to 3 (0 is negative, 1 is low, 2 is medium, and 3 is high). All statistical significance was obtained through *z*-score calculator for 2 population proportions (https://www.socscistatistics.com/tests/ztest/) between a group with scores 0 and 1(negative to low intensity) and a group with scores 2 and 3 (medium to high intensity).

### Analysis of ACAA2 mRNA levels in patient datasets

*ACAA2* mRNA levels across various cancer cell lines were assessed via Cancer Cell Line Encyclopaedia (CCLE) [34]. *ACAA2* mRNA levels were also assessed from 5 independent, previously published datasets: 3 for prostate cancer patients [35–37] and 2 for lung cancer patients [38, 39]. Data were accessed via cBioPortal for Cancer Genomics (https://www.cbioportal.org). The *ACAA2* mRNA expression *z*-scores relative to all samples were obtained from the datasets, and data analysis was performed via Prism 9.0 software. Student’s *t*-test was performed to compare the *ACAA2* mRNA levels in these two groups. For the Abida et al. (2019) dataset, AR scores and NEPC scores were used to sort patients into NEPC and CRPC groups [36]. The top 30 patients with the highest NEPC score and the lowest AR score were determined into the NEPC group, and the top 30 patients with the lowest NEPC score and the highest AR score were characterised into the adeno-CRPC group (Supplementary Fig. S1C). *ACAA2* mRNA *z*-scores between lung adenocarcinoma and SCLC in the Rohrbeck et al. (2008) dataset, and *ACAA2* mRNA expression between normal lung, lung adenocarcinoma, and SCLC were compared in the Bhattacharjee et al. (2001), dataset. All patient groups were assumed to follow a normal distribution. Information on patient exposure to platinum-based therapy was also utilised from the Rohrbeck et al. (2008) dataset. Samples where ACAA2 mRNA expression information is not available are excluded from the analysis.

### Overall survival

Kaplan–Meier curve in lung adenocarcinoma was plotted with data from KM Plotter (https://kmplot.com/analysis/) [40]. Kaplan–Meier curve in neuroblastoma was generated via Prism 9.0. Patient overall survival and ACAA2 mRNA expression were obtained from Target, 2018 (phs000467.v21.p8) through cBioPortal for Cancer Genomics. Hazard ratio with confidence intervals and Log-rank *P*-values (Mantel-Cox test) were calculated. ACAA2 high and low groups were sorted using the median ACAA2 expression as the threshold for cut-off.

### Statistical analysis

When comparing the statistical significance between two groups, Student’s *t*-test was performed on Prism 9.0 software. All *t*-tests were two-tail, unpaired, and parametric, and *P*-values of 0.05 or less were considered statistically significant (*P  <  0.05, **P  <  0.01, ***P  <  0.001, ****P  <  0.0001, ns = non-significant). Significance levels for contingency plots were obtained as *z*-score for 2 populations proportions via online calculators (socscistatistics.com).

## Results

### ACAA2 protein level is elevated in NEPC

**Fig. 1:**
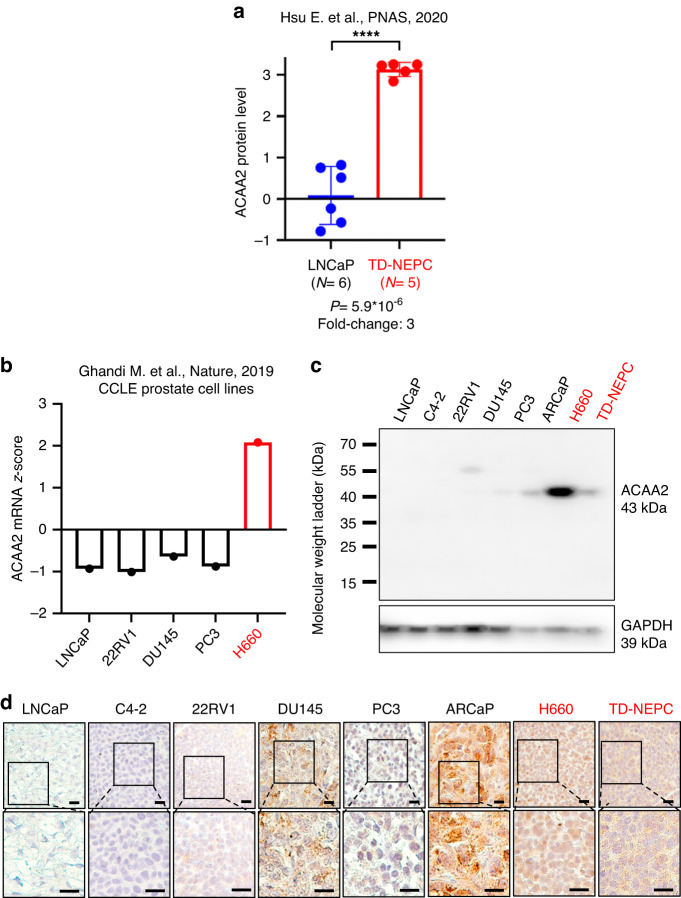
ACAA2 expression is elevated in NEPC. **a** Protein level of ACAA2 is significantly elevated in NEPC tumours relative to the LNCaP control tumours via proteomic analysis that compares ACAA2 before and after the onset of NE phenotype in LNCaP and TD-NEPC. Both control LNCaP and TD-NEPC tumours were generated (*N* = 6 for LNCaP and *N* = 5 for NEPC) for proteomics. Student’s *t*-test was used, mean ± SD, ****P  <  0.0001. **b** *ACAA2* mRNA is increased in the NEPC H660 cell line relative to all other prostate cancer cell lines. Comparison of *ACAA2* mRNA expressions across prostate cancer cell lines. *ACAA2* mRNA *z*-scores were obtained from CCLE (Cancer Cell Line Encyclopaedia) [34] via cBioportal (http://www.cbioportal.org). **c** Western blot (WB) analysis of prostate cancer cell lines. Cell lines with NE features are highlighted in red. **d** Immunohistochemistry (IHC) staining of ACAA2 protein expression across various prostate cancer cell line xenografts shows that ACAA2 expression is notably increased in DU145, ARCaP, H660, and TD-NEPC. Images were taken using Leica microscope, and the scale bar represents 20 microns (upper) and 10 microns (lower), respectively.

ACAA2 expression was significantly elevated (*P* = 5.9*10^-6^) in TD-NEPC tumours with a fold-change of 3 (Fig. 1a) relative to the control, non-SCNC LNCaP tumours in analysis of previously published proteomic profiling [26]. *ACAA2* mRNA expression was also elevated in the NEPC cell line, NCI H660, relative to non-SCNC prostate cancers when assessing cell line mRNA levels using data from the Cancer Cell Line Encyclopaedia (CCLE) (Fig. 1b) [34]. To assess the correlation of ACAA2 with prostate cancer malignancy, western blot (WB) was performed to assess ACAA2 protein expression (Fig. 1c). We also generated tumour xenografts from the same cell lines and performed immunohistochemistry (IHC) analysis. WB and IHC results indicated that ACAA2 protein level positively correlated with SCNC phenotypes as ACAA2 was highly expressed in both NEPC strains (H660 and TD-NEPC) (Fig. 1c, d). ACAA2 was also expressed in DU145 and PC3 xenografts, both of which exhibit AR loss and are, therefore, resistant to androgen deprivation therapies (Fig. 1c, d). DU145 has recently been characterised as double-negative prostate cancer (DNPC), which, like NEPC, also stems from CRPC and occurs in 20% to 15% of metastatic CRPC cases [15]. DNPC is highly aggressive due to enhanced metastasis and stemness signature, and treatment options remain limited [15]. We also observed that ACAA2 was expressed in ARCaP, which is an AR-low, androgen-repressed prostate cancer cell line that exhibits increased metastatic potential to bones [41, 42]. WB and IHC results consistently demonstrated high ACAA2 expression in the NEPC cell lines H660 and TD-NEPC (Fig. 1c, d). In addition, increased *ACAA2* expression also corresponded with increases in standard NE markers, SYP, CD56, and CHGA, in two published mCRPC datasets (Supplementary Fig. S1A, B). In both the Abida et al. (2019) and the Nguyen H. et al. (2017) datasets, our results demonstrated a significant positive correlation between *ACAA2*, *SYP* (*P* = 0.0002, 0.0044), *CD56* (*P* = 0.001), and *CHGA* (*P* = 0.0001, 0.0087) (Supplementary. Fig. S1A, B). These results suggest an association between ACAA2 expression and the NE phenotype.

### High levels of ACAA2 correlate with prostate cancer progression in patient samples

**Fig. 2:**
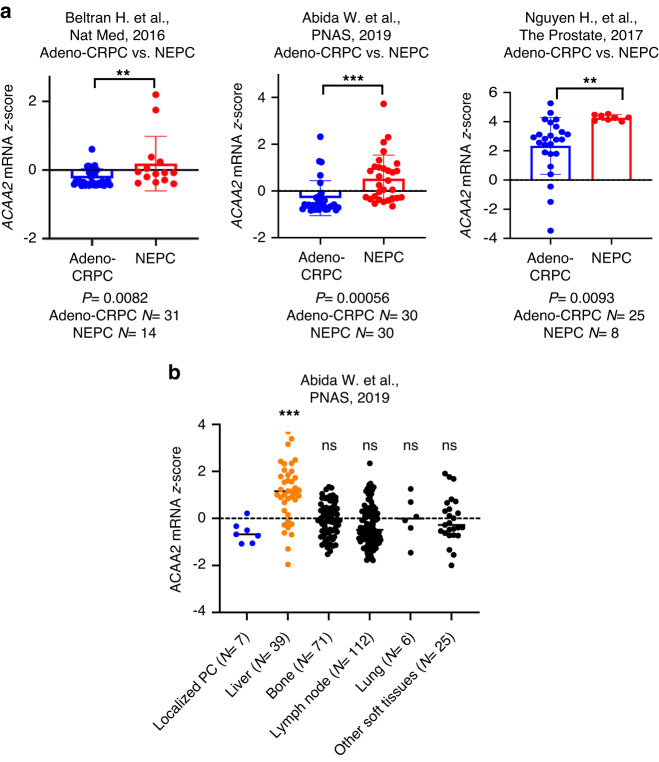
*ACAA2* mRNA expression correlates with NEPC patients in multiple patient datasets. **a** *ACAA2* mRNA expression is higher in NEPC patients when compared to adeno-CRPC patients. Three independent prostate cancer patient datasets, Beltran et al. (2016) [35], Abida et al. (2019) (SU2C/PCF) [36], and Nguyen et al. (2017) [37], were used to analyse *ACAA2* mRNA *z*-scores in prostate cancer patients. *P*-values and sample sizes are included at the bottom of each box plot. **b** Information regarding metastatic sites was also obtained from the Abida et al. (2019) (SU2C/PCF) [36] dataset. Patient samples were then sorted based on the metastatic site, and each group’s *ACAA2* mRNA expression levels were compared to localised prostate cancer. Student’s *t*-test was used across all analyses, mean ± SD, ns = non-significant, *P  <  0.05, **P  <  0.01, ***P  <  0.001, ****P  <  0.0001.

To validate the positive correlation between *ACAA2* expression and the NE phenotype, we analysed three published patient datasets via cBioPortal, and compared *ACAA2* mRNA expression in NEPC and adeno-CRPC tumours. *ACAA2* levels were significantly elevated in NEPC patient groups relative to adeno-CRPC patient groups from all three datasets, including Beltran et al. (2016) (*P* = 8.2*10^-3^), Abida et al. (2019) (*P* = 5.6*10^-4^), and Nguyen et al. (2017) (*P* = 9.3*10^-3^) (Fig. 2a and Supplementary Fig. S1B, C). These results implicated ACAA2 as a candidate molecular indicator for the SCNC phenotype and suggested a positive association between ACAA2 expression and prostate cancer progression. In addition, patient samples from the Abida (2019) [36] dataset were sorted based on the metastatic site, and samples from each site were then compared to localised prostate cancer samples. There was a significant increase of *ACAA2* mRNA expression in samples from liver metastasis when compared to samples from localised prostate cancer (Fig. 2b). Studies have shown that the prevalence of liver metastasis is increased in NEPC patients and correlates with reduced overall survival in CRPC patients [43]. The correlation between ACAA2 expression and increased liver metastasis further suggests its association with the aggressive clinical course observed in NEPC patients.

**Fig. 3:**
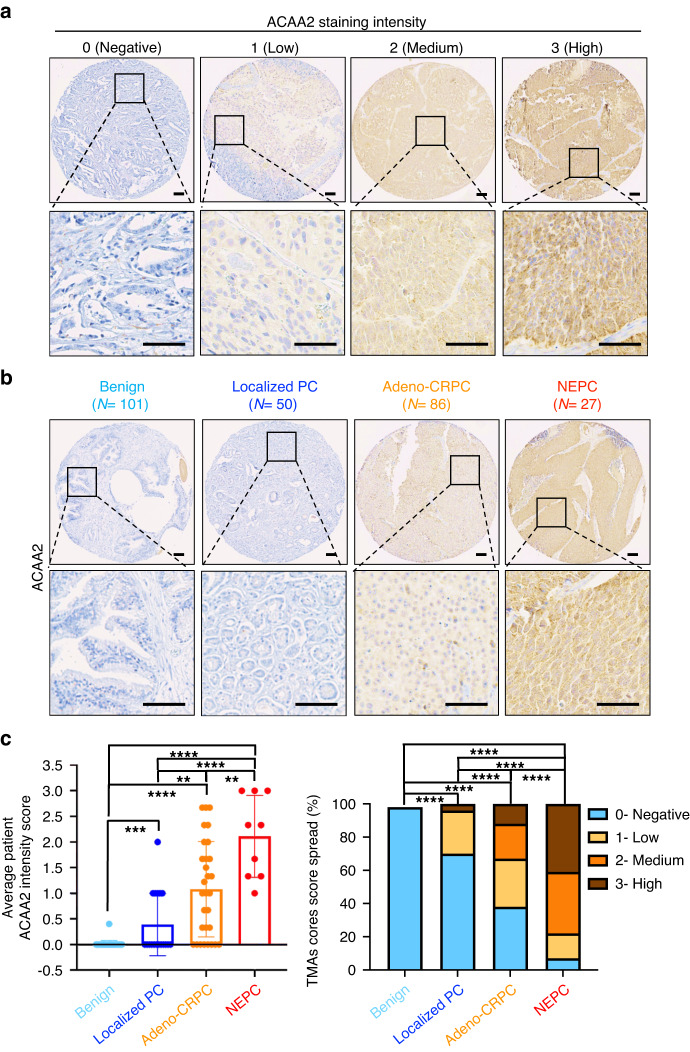
ACAA2 protein expression is positively correlated with prostate cancer progression and malignancy. **a** To validate previous findings in patient samples, two prostate cancer TMAs were stained for ACAA2 and scored on a scale from 0 to 3 (0 represents negative, 1 represents low intensity, 2 represents median intensity, and 3 represents high intensity). The first TMA contains cores from benign prostate (*N* = 101) and localised prostate cancer (*N* = 50), and the second TMA is composed of tissue cores from NEPC (*N* = 86) and adeno-CRPC (*N* = 27). Scale bars represent 5 microns (upper) and 20 microns (lower) respectively. **b** Representative images from each group of prostate cancer progression: benign, localised prostate cancer, adeno-CRPC, and NEPC. Images were captured via Leica microscope, and scale bars represent 5 microns (upper) and 20 microns (lower), respectively. **c** Averaged ACAA2 staining intensity by patient and ACAA2 staining score spread. The dot plot on the left panel shows the score distribution across the spectrum of prostate cancer advancement. Each dot represents the averaged staining score of all cores obtained from a patient (35 patients with benign prostate; 18 patients with localised PC; 29 patients with adeno-CRPC; 9 patients with NEPC). The contingency plot on the right illustrates the percentage of each score amongst all sample cores within each prostate cancer group, including benign, localised, adeno-CRPC, and NEPC. Student’s *t*-test was performed, mean ± SD, and **P  <  0.01, ***P  <  0.001, ****P  <  0.0001.

To assess the profile of ACAA2 expression in prostate cancer tumours, we stained patient tissue microarrays (TMAs) for ACAA2 (Fig. 3a, b). Two TMAs were utilised: one containing benign prostate cores and localised prostate cancer cores (35 patients/101 cores with benign prostate; 18 patients/50 cores with localised prostate cancer), and another containing adeno-CRPC and NEPC PDX cores (29 PDX/ 86 cores with adeno-CRPC; 9 PDX/27 cores with NEPC). IHC revealed that ACAA2 was selectively expressed in cancerous cores relative to benign cores (Supplementary Fig. S2). Only 2 out of the 101 (1.98%) normal prostate cores stained positive (staining intensity score >= 1) for ACAA2 while 93 of the 163 (57%) prostate cancer cores (including localised, adeno-CRPC, and NEPC) stained positive for ACAA2 (*P* < 10^-4^), demonstrating ACAA2’s selective elevation in cancerous phenotypes (Supplementary Fig. S2). Importantly, ACAA2 staining from PDX TMAs of NEPC and adeno-CRPC tumours revealed an increase in ACAA2 protein levels in NEPC when compared to adeno-CRPC PDXs (Fig. 3b–c). Only 33% (28 out of 86) of adeno-CRPC cores stained with medium/high intensity (intensity scores of 2 and 3) for ACAA2, while 78% (21 out of 27) of NEPC cores stained medium/high for ACAA2 (Fig. 3c). None of the benign prostate tissues, only 2 out of 50 (4%) localised prostate cancer samples, and 10 out of 86 (12%) in adeno-CRPC stained high for ACAA2, which is significantly lower than the 11 out of 27 (41%) of NEPC samples (Fig. 3c). This indicates that ACAA2 level is upregulated in patients with more advanced prostate cancers and positively correlates with prostate cancer progression. IHC results also indicate that protein expression of ACAA2 positively associates with protein levels of NE markers: CHGA (*P* = 3.95*10^-2^) and SYP (*P* = 8.3*10^-3^), which further supports the correlation between elevated ACAA2 protein expression and the SCNC phenotype (Supplementary Fig. S3).

### ACAA2 expression is elevated in other SCNCs

**Fig. 4:**
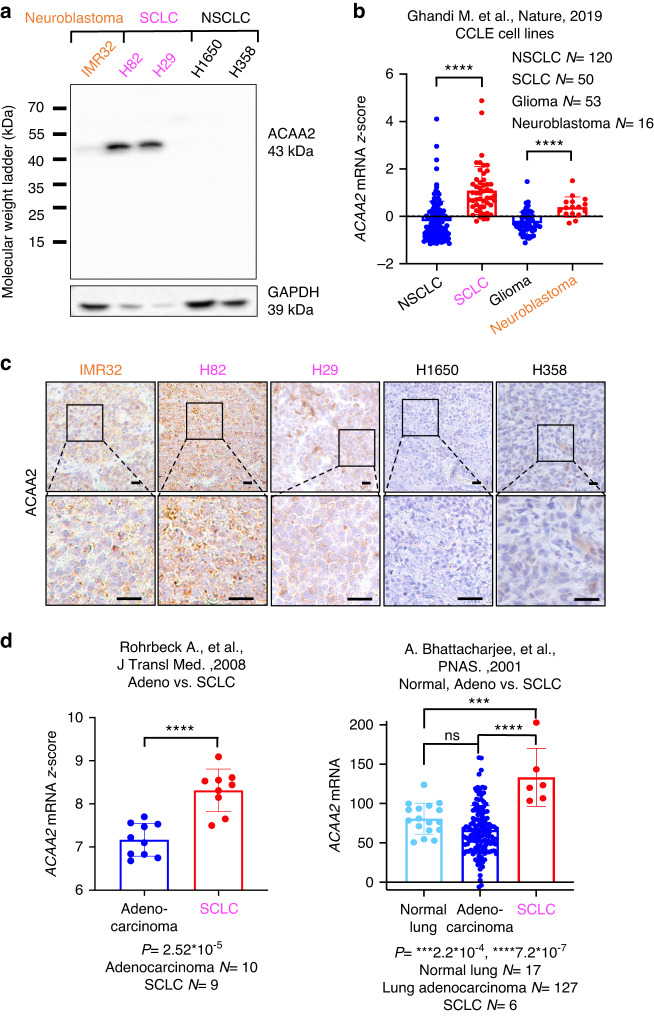
ACAA2 expression correlates with NE phenotype in other SCNCs such as SCLC and neuroblastoma. **a** Western Blot of ACAA2 expression in neuroblastoma (IMR32), SCLC (H82, H29), and NSCLC (H1650, H358) cell lines. **b** *ACAA2* mRNA *z-*score analysis in NSCLC, SCLC, glioma, and neuroblastoma cell lines from the CCLE dataset [34]. **c** IHC of ACAA2 protein levels in neuroblastoma, SCLC, and NSCLC cell line xenografts. Scale bar represents 20 microns (upper) and 10 microns (lower), respectively. **d** *ACAA2* mRNA expression in SCLC relative to the non-SCNC lung adenocarcinoma and normal lung tissue. Utilising the Rohrbeck [38] and the Bhattacharjee [39] datasets, *ACAA2* mRNA expression in SCLC were compared against lung adenocarcinoma and normal lung samples. Student’s *t*-test was performed, mean ± SD, and the *P*-value and sample size are included beneath each dataset (*P  <  0.05, **P  <  0.01, ***P  <  0.001, ****P  <  0.0001, ns =  non-significant).

We identified that ACAA2 protein expression is also elevated in NE cell lines IMR32 (neuroblastoma), H82 (SCLC), and H29 (SCLC) measured by WB (Fig. 4a). All non-small-cell lung cancer (NSCLC) cell lines, including H1650 and H358, exhibited undetectable levels of ACAA2 by WB (Fig. 4a). We also assessed *ACAA2* mRNA levels in CCLE [34] to demonstrate that *ACAA2* mRNA expression is also significantly increased in SCLC (*N* = 50) and neuroblastoma cell lines (*N* = 16), both of which are NE cancers, relative to their non-NE counterparts: NSCLC (*N* = 120) and glioma (*N* = 53) (Fig. 4b and Supplementary Fig. S4A, B). While glioma is not derived from the same tissue of origin as neuroblastoma, it has been used as a non-NE comparator for neuroblastoma [19, 44]. When compared to glioma, *ACAA2* expression level in neuroblastoma was significantly elevated (*P* = 2.5*10^-6^) (Fig. 4b). The same trend was observed in SCLC relative to NSCLC with *P* = 5.7*10^-15^ (Fig. 4b). Then, IHC was performed on neuroblastoma, SCLC, and NSCLC tumour xenografts and demonstrated enhanced expression of ACAA2 in neuroblastoma and SCLC relative to NSCLC (Fig. 4c).

The correlation between ACAA2 and the SCNC phenotype was further confirmed via *ACAA2* mRNA level analysis in two independent lung cancer patient datasets (Fig. 4d) [38, 39]. SCLC exhibited significantly elevated *ACAA2* mRNA expressions when compared to lung adenocarcinoma [*P* = 2.52*10^-5^ (left panel); 7.2*10^-7^ (right panel)] and normal lung tissues (*P* = 2.2*10^-4^), which further suggests a positive correlation between ACAA2 expression and SCNCs (Fig. 4d). In addition, consistent with the relationship observed in prostate cancer, *ACAA2* mRNA expression also positively correlated with expressions of CHGA and CD56 (Supplementary Fig. S5). In Rohrbeck et al. (2008) dataset, increased ACAA2 correlated with the SCLC phenotype relative to lung adenocarcinoma as well as an increased expression of NE markers CHGA (*P* = 9.5*10^-3^) and CD56 (*P* = 3.81*10^-2^) (Supplementary Fig. S5A). This is also observed in the Bhattacharjee et al. (2001) dataset, with *P* < 10^-4^ for both positive correlations between ACAA2, CHGA, and SYP (Supplementary Fig. S5B).

**Fig. 5:**
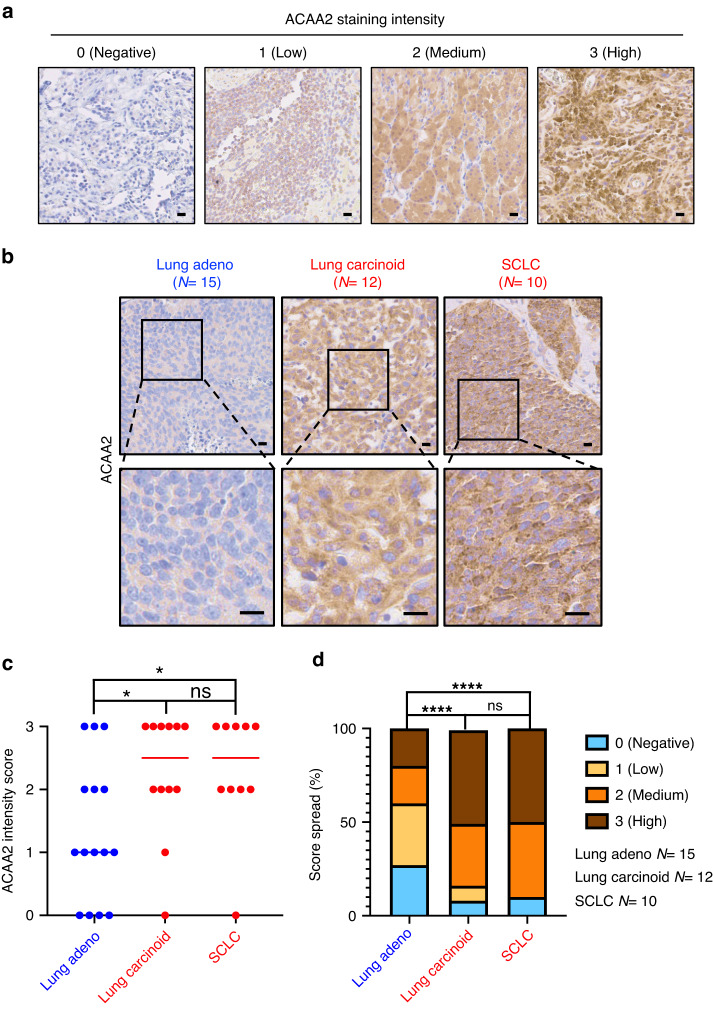
ACAA2 is elevated in lung patient tumour tissues. **a** Scoring guideline for ACAA2 staining intensity. IHC was performed on patient tissues ranging from lung adenocarcinoma (*N* = 15) to the NE lung carcinoid (*N* = 12) and SCLC (*N* = 10). The staining was scored on an intensity scale from 0 to 3. A score of 0 represents negative ACAA2; a score of 1 represents low ACAA2 intensity; a score of 2 represents medium ACAA2 intensity; and a score of 3 represents high ACAA2 intensity. **b** Representative IHC images from lung adenocarcinoma, lung carcinoid, and SCLC. ACAA2 expression is elevated in the NE groups, including lung carcinoid and SCLC (in red), relative to the lung adenocarcinoma (in blue). Scale bar represents 5 microns (upper) and 20 microns (lower), respectively. **c** Scoring distribution in lung adenocarcinoma, lung carcinoid, and SCLC. ACAA2 expression increases in lung carcinoid (*P* = 0.033) and SCLC (*P* = 0.034) relative to lung adenocarcinoma. Student’s *t*-test was performed with *P  <  0.05, ns =  non-significant. **d** Contingency plot that displays the percentage of each score across all samples in each patient groups. 40% (6/15) of lung adenocarcinoma stained with medium/high intensity (intensity score > = 2) while 83% (10/12) of lung carcinoid (*P* = 10^-5^) and 90% (9/10) of SCLC stained medium/high intensity (*P* = 10^-5^). *P*-value is calculated as *z*-score for 2 populations proportions. ****P  <  0.0001, ns = non-significant.

To assess the clinical relevance of the correlation between ACAA2 and SCNC phenotypes, IHC staining of ACAA2 in lung adenocarcinoma patient tumour (*N* = 15) was compared to ACAA2 staining in patient samples from lung carcinoid tumour (*N* = 12) and SCLC (*N* = 10), both of which exhibit NE phenotypes (Fig. 5a–d). Using a 0 to 3 intensity scale (Fig. 5a), we discovered an increase of ACAA2 expression in lung carcinoid (*P* = 0.033) and SCLC (*P* = 0.034), relative to the non-NE, adenocarcinoma patient samples (Fig. 5b, c). The elevation of ACAA2 in NE lung cancers was further confirmed in the contingency plot of intensity scores (Fig. 5d). Compared to lung adenocarcinoma where 40% (6/15) of cases stained with medium/high intensity, 83% (10/12) of lung carcinoid tumours stained with medium/high intensity (*P* = 10^-5^) (Fig. 5d). The same trend was observed in SCLC, where 90% (9/10) of SCLC stained with medium/high intensity (Fig. 5d). This elevation suggests a correlation between ACAA2 levels and NE features in clinical patient samples. To explore ACAA2’s expression in other tumour types with NE features, we stained ACAA2 in a TMA (NE921) with 20 tumours with NE features, 16 adenocarcinomas, and 8 normal cases (Supplementary Fig. S6). While ACAA2 was detected in cases with NE features, there was no significant difference in ACAA2 intensity relative to normal and adenocarcinoma groups (Supplementary Fig. S6). This could be the result of a small number of samples or differences in primary sites. Further exploration in larger cohorts will be necessary to validate the trend and to elucidate the differences between ACAA2 expression in various primary sites.

**Fig. 6:**
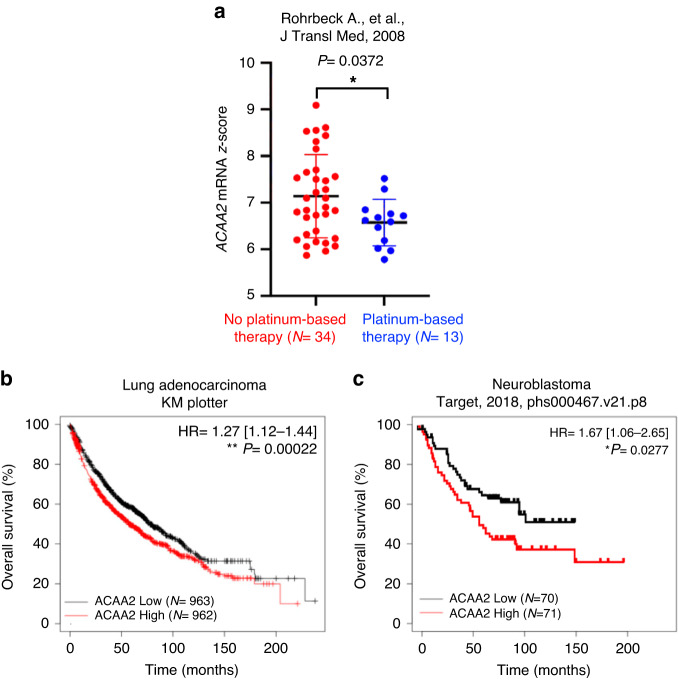
High levels of ACAA2 are associated with shorter overall survival. **a** *ACAA2* mRNA expression in lung cancer patients without exposure to platinum-based relative to those treated with platinum-based therapy. Student *t*-test was performed with *P  <  0.05. **b** Kaplan–Meier plot of overall survival in lung cancer patients comparing the patient outcomes of ACAA2 high and ACAA2 low groups. ACAA2 high group contains *N* = 962 and ACAA2 low has *N* = 963. **c** Kaplan–Meier plot of the overall survival in neuroblastoma patients with high and low *ACAA2* mRNA expression. Patients were split by median ACAA2 expression into ACAA2 high (*N* = 71) and ACAA2 low (*N* = 70) groups. HR = hazard ratio with confidence intervals, and Log-rank *P*-values were computed with *P  <  0.05 and **P  <  0.01.

Lastly, we assessed whether increased ACAA2 expression is associated with patient clinical prognosis. We discovered that ACAA2 expression is lower in lung cancer patients who were exposed to platinum-based therapy relative to non-treated patients, and that increased ACAA2 was associated with worse overall survival in lung adenocarcinoma (*P* = 2.2*10^-4^) and neuroblastoma patients (*P* = 2.77*10^-2^) (Fig. 6). Based on these results, we hypothesise that increased ACAA2 is associated with worse clinical outcome. Exploration of this association in additional patient cohorts to elucidate the clinical potential of ACAA2 will be critical to the translation of this knowledge into clinics.

## Discussion

Based on our result, we hypothesise that ACAA2 has the potential to differentiate cancers with NE features from their non-NE counterparts in SCNCs like NEPC and SCLC. In addition, our study suggests the role of ACAA2 in prostate cancer disease progression and its potential as a therapeutic target for advanced, metastatic prostate cancers.

SCNCs exhibit similar molecular profiles and genetic alterations, suggesting a common pathway of transdifferentiation in multiple cancers [1, 4, 5]. Published studies have shown that converging genetic expression profiles are observed as cancers progress into the SCNC phenotype [4]. For instance, loss of REST transcriptional repression, increased EZH2, amplified MYCN, loss of TP53, and loss of RB1 have been suggested to foster the SCNC phenotype across multiple tissue sites [35, 45, 46].

Currently, the survival rate for patients with SCNCs remains low due to their therapy resistance and the extensive metastasis that is a hallmark of SCNCs and contributes to its worse clinical outcome [4]. The platinum and etoposide-based chemotherapies that form the frontline of SCNC treatments show short-term clinical effects on patient survival [1, 4, 5]. As a result, there is an urgent need to identify pathways crucial for NE transdifferentiation in order to discover novel therapeutic targets to broaden the treatment options for this lethal disease.

Since ACAA2 is overexpressed in various types of SCNCs and is also upregulated by Trop2-driven NE transdifferentiation, ACAA2 might play a role in NE progression to SCNCs. ACAA2 is a key enzyme in the mitochondrial fatty acid beta-oxidation pathway [27–29]. As ACAA2 is consistently elevated in NEPC and SCLC, we propose the possibility of a linkage between ACAA2, fatty acid beta-oxidation, and NE progression. Thus, the inhibition of ACAA2 may have therapeutic benefits in NE cancers. In fact, a previous study suggested a shared sensitivity towards the disruption of lipid metabolism in SCNCs [4]. Currently, there is a pharmacological ACAA2 inhibitor, trimetazidine hydrochloride, that is currently used to treat angina pectoris and myocardial ischaemia as an anti-ischaemic (anti-anginal) metabolic agent in Europe [47]. As an identified fatty acid oxidation inhibitor, trimetazidine was shown to elevate myocardial glucose metabolism and to increase glucose level during ischaemia through the inhibition of fatty acid metabolism [47–49]. Various studies have also demonstrated that the inhibition of fatty acid beta-oxidation via trimetazidine enhances glucose oxidation [47, 50, 51]. Our study supports further exploration of this ACAA2 inhibitor to test its therapeutic efficacy against NEPC and SCLC.

Our study demonstrates that ACAA2 expression is elevated in cancers with NE phenotype through assessing the expression profile of ACAA2 in cell lines and xenografts of neuroblastoma as well as cell lines, xenografts, patient mRNA, and patient tissue samples from prostate and lung cancers. This study supports further assessment of ACAA2 expression profile in larger, independent patient cohorts across various types of SCNCs to further delineate its candidacy as a molecular indicator for SCNCs and a potential therapeutic target.

### Supplementary information

Supplementary Figures and Figure Legends

## Data Availability

All data used in this publication can be found in the methods section, figures, or supplementary information.
