# Supplementary material for: ACAA2 is a novel molecular indicator for cancers with neuroendocrine phenotype
Source: Br J Cancer. 2023 Oct 5;129(11):1818–28. doi: 10.1038/s41416-023-02448-y (PMC10667239; doi:10.1038/s41416-023-02448-y)
Supplement: Supplementary file 1 — Supplementary Figures and Figure Legends [file 41416_2023_2448_MOESM1_ESM.pdf]

Supplementary Figure S1

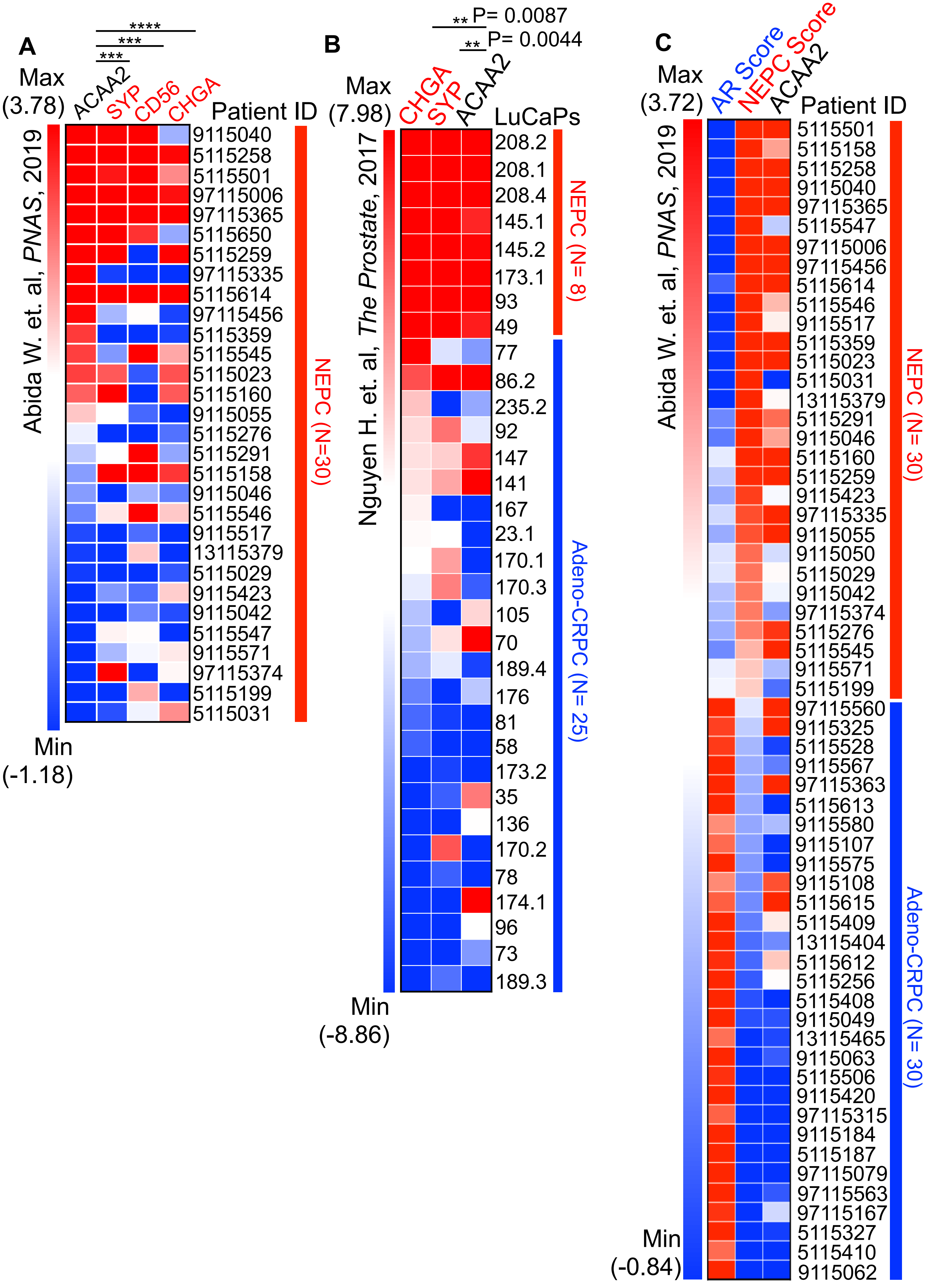

Supplementary Figure S1. *ACAA2* mRNA Levels Correlates with NE Features in Patients Samples.

(**A**) *ACAA2* mRNA level is elevated in patients with high NEPC score and low AR score, correlating with the NE phenotype. Utilizing cBioPortal to access AR score, NEPC score, and *ACAA2* mRNA z-scores from Wassim A. et. al, *PNAS*, 2019[1], samples were sorted into 2 groups. The top 30 patients with the highest NEPC Scores and the lowest AR scores were classified as NEPC, and this group expresses high *ACAA2* mRNA levels. On the other hand, the 30 patients with the lowest NEPC score and the highest AR scores correlate with non-NE phenotypes and exhibit reduced *ACAA2* mRNA expression. The Patient ID from each sample is labeled accordingly. (**B**) Heat map shows correlation between *ACAA2* mRNA expression profile and that of standard NE markers, *SYP*, *CD56*, and *CHGA* in the 30 NEPC samples as described above. Correlation analyses were performed with ***P< 0.001 and ****P<0.0001. Corresponding Patient IDs are labeled accordingly. (**C**) *ACAA2* mRNA expression in a patient-derived-xenograft model (PDX) with NEPC and adeno-CRPC patients. Data obtained from Nguyen H. et. al, *The Prostate*, 2017[2] and contains the *CHGA*, *SYP*, and *ACAA2* mRNA expressions from NEPC and adeno-CRPC patients. CHGA and SYP are NE markers, and elevated CHGA and SYP correlates with NEPC features. As shown in the heatmap, *ACAA2* mRNA expression is elevated in NEPC patients with high NEPC markers (high CHGA and SYP) relative to adeno-CRPC patients. Correlation analyses were performed with **P<0.01. NEPC and adeno-CRPC designations are the same as that characterized in Nguyen H. et. al, *The Prostate*, 2017[2]. The LuCaPs series are labeled accordingly.

Supplementary Figure S2

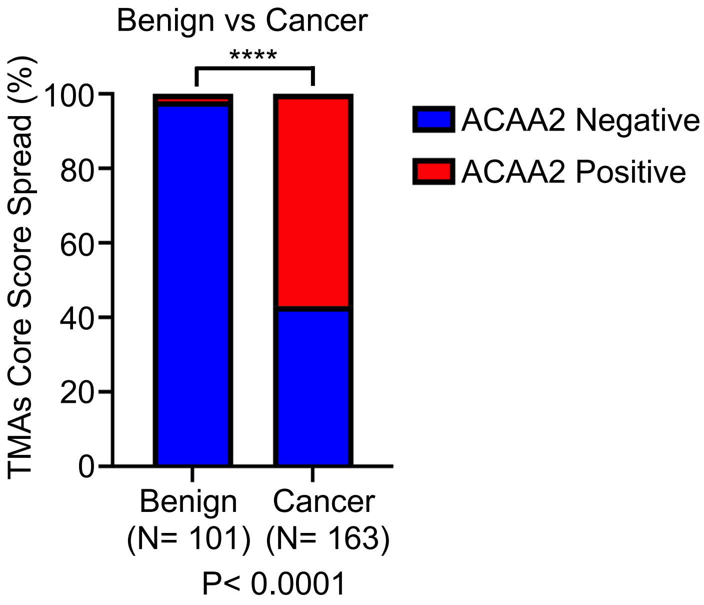

Supplementary Figure S2. ACAA2 is Specifically Expressed in Prostate Cancer TMAs Samples Relative to Benign Samples.

(A) IHC was performed to assess the ACAA2 expression profile in TMAs samples. The contingency plot represents the percent of cores that were ACAA2 negative (in blue) and ACAA2 positive (in red). ****P<0.0001.

Supplementary Figure S3

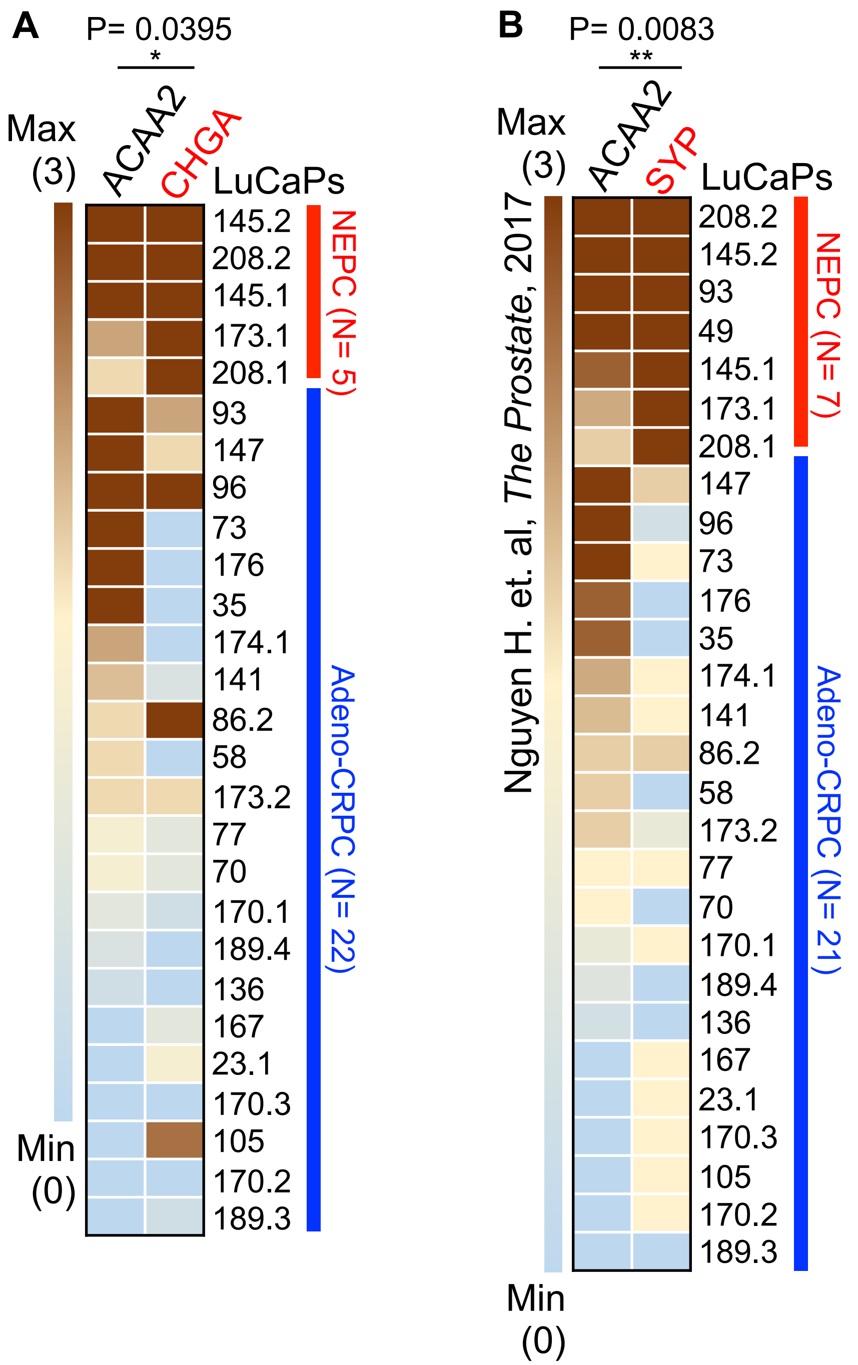

Supplementary Figure S3. LuCaPs PDX TMA Shows Protein Correlation between ACAA2 and Standard NE Markers, CHGA and SYP.

(**A**) Heat map shows the CHGA and ACAA2 protein expressions for each LuCaPs PDX model. A score of 3 corresponds to high staining intensity, with 2 representing medium, 1 representing low, and 0 representing negative expression. The intensities for each PDX model were obtained by averaging the IHC results of the triplicate cores on the LuCaPs TMA. Cores that were lost or damaged during IHC were not included. Correlation analysis was performed with *P<0.05. The corresponding LuCaPs PDX models are labeled accordingly. (**B**) Heat map representation of the ACAA2 and SYP protein expression in LuCaPs PDX models. The SYP IHC protein expression profile was obtained from Nguyen H. et. al, *The Prostate*, 2017 and matched to the corresponding ACAA2 TMA staining[2]. Correlation analysis was performed with **P<0.01.

Supplementary Figure S4

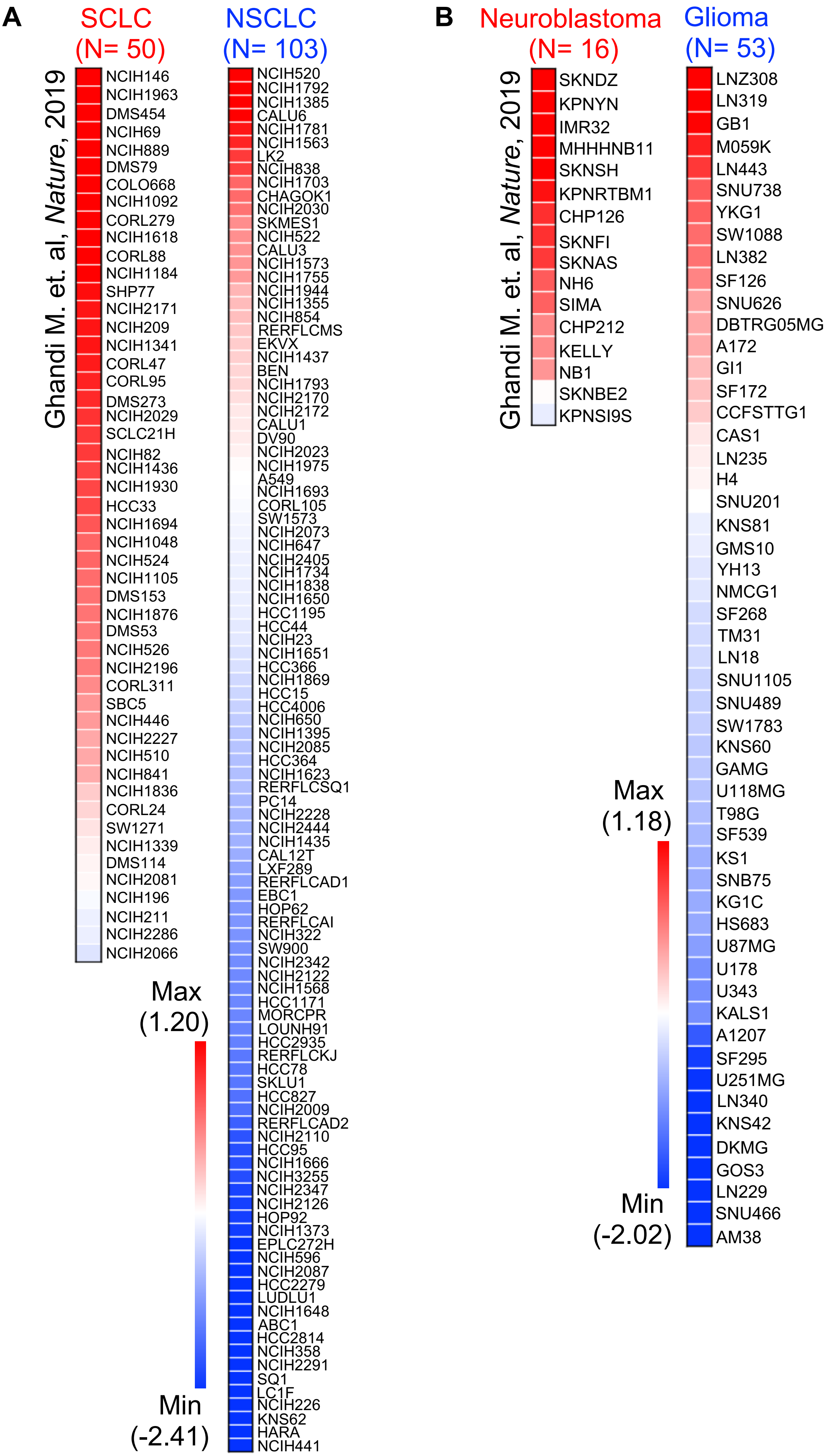

Supplementary Figure S4. *ACAA2* mRNA Levels Correlates with NE Features in SCLC and Neuroblastoma Cell Lines.

(**A**) *ACAA2* mRNA expression levels from SCLC (N= 50) and NSCLC (N= 103) cell lines are plotted as heat maps for comparison. The name of all cell lines plotted are labeled accordingly. (**B**) *ACAA2* mRNA profile was also obtained from the neuroblastoma (N= 16) and its non-NE counterpart, glioma (N= 53) cell lines. The heat map shows increased *ACAA2* expression in neuroblastoma cell lines relative to glioma cell lines. Data obtained from CCLE (Ghandi M. et. al, *Nature*, 2019[3]) and all cell lines are labeled next to the representing datapoint on the heat map.

Supplementary Figure S5

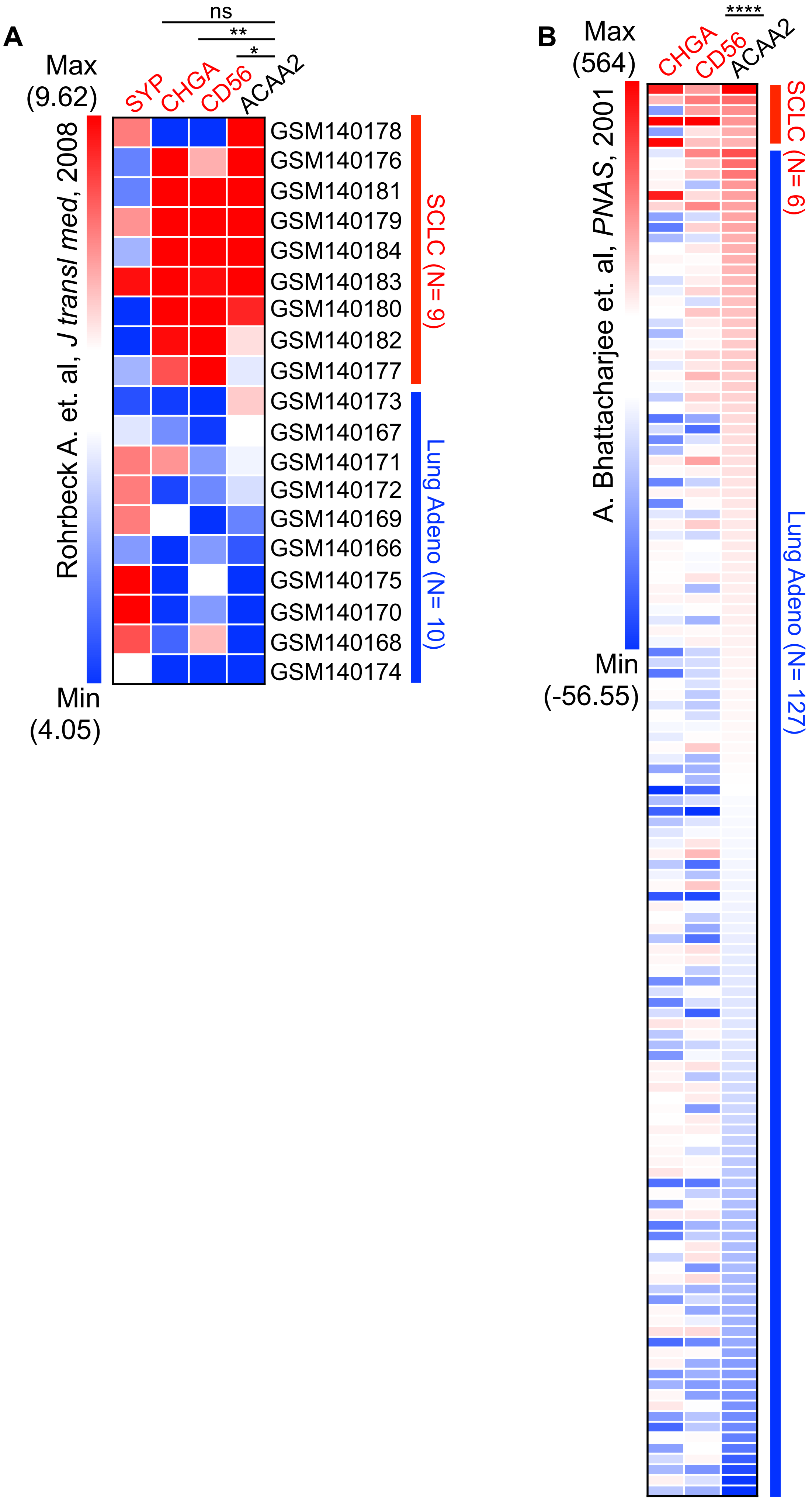

Supplementary Figure S5. ***ACAA2*** **mRNA Expression Correlates with NE Features in Lung Cancer.**

(**A**) Heat map represents the association between *ACAA2*, *SYP*, *CHGA*, and *CD56*. The mRNA z-scores were obtained from the Rohrbeck et. al., *J transl med*, 2008[4] dataset. Correlation analyses were performed with ns= non-significant, *P< 0.05, **P<0.01. Corresponding sample IDs are labelled accordingly. (B) Heat map reflects the correlation between *ACAA2* mRNA expression and the NE phenotype in lung cancer samples (SCLC N= 6, adenocarcinoma N= 127) obtained from A. Bhattacharjee et. al., *PNAS*, 2001[5]. Correlation analysis was performed with ****P<0.0001. *SYP* mRNA expression was not profiled in this dataset.

Supplementary Figure S6

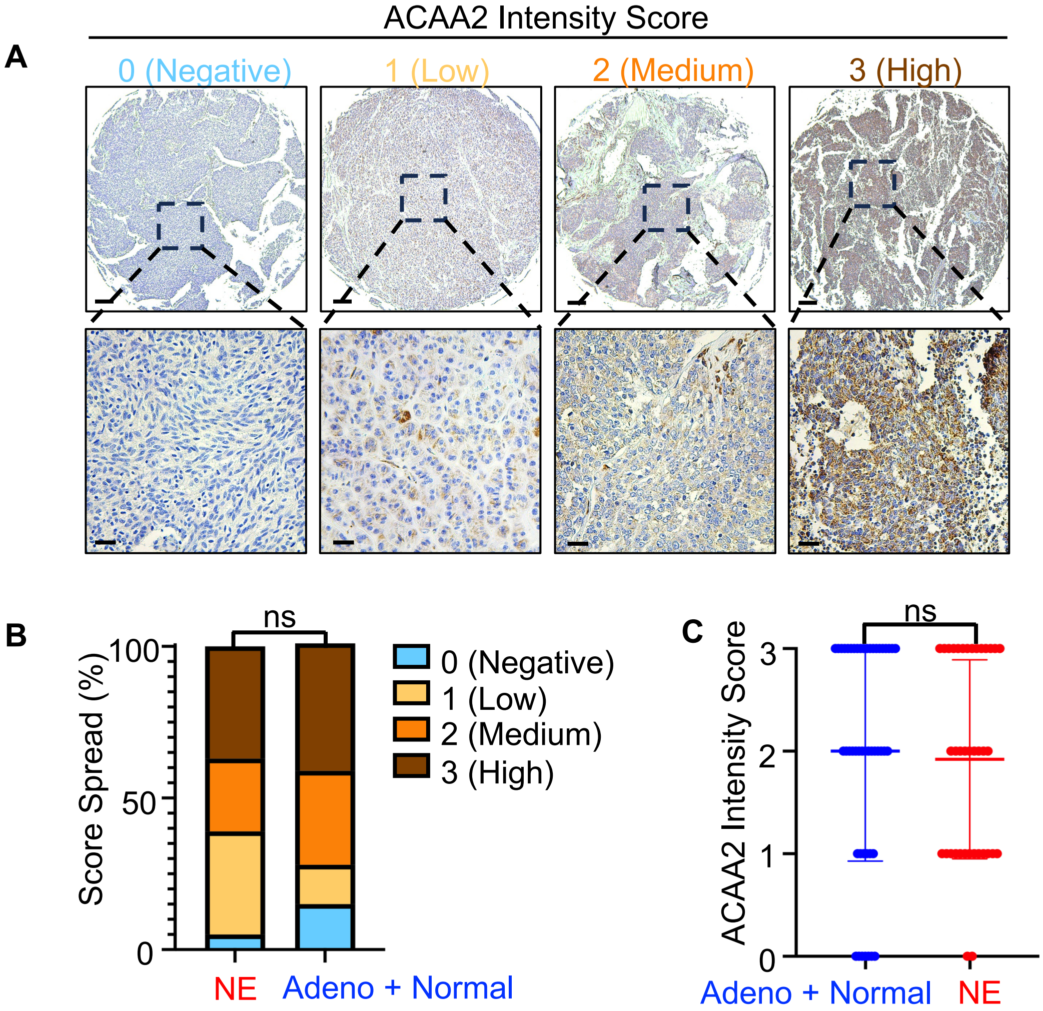

Supplementary Figure S6. Immunohistochemical Analysis of ACAA2 in Additional Cancers with Neuroendocrine Phenotype.
(**A**) ACAA2 Intensity scale used to score ACAA2 IHC staining in the NE921 TMA with 20 NE neoplasms from 9 organs (mediastinum, cardia, gallbladder, colon, small intestine, pancreas, rectum, stomach, and lung), 16 adenocarcinomas, and 8 normal tissues. Each case has 2 cores on the TMA. Score of 0 is negative staining, 1 is low intensity, 2 is medium intensity, and 3 is high intensity. Scale bars represent 20 microns and 4 microns respectively. (**B**) Contingency plot shows the score spread percentage of each score in cancer with neuroendocrine features (N= 20) relative to the adenocarcinoma and normal group (N= 24). Z-score calculations for 2 population proportions were performed with ns= non-significant. (**C**) ACAA2 intensity score spread of the TMA cores in the 2 groups. Student t-test was performed with ns= noon-significant.
